# The role of the chemokines MCP-1, GRO-α, IL-8 and their receptors in the adhesion of monocytic cells to human atherosclerotic plaques

**DOI:** 10.1016/j.cyto.2008.05.009

**Published:** 2008-08

**Authors:** Charikleia Papadopoulou, Valerie Corrigall, Peter R. Taylor, Robin N. Poston

**Affiliations:** a Centre for Cardiovascular Biology and Medicine, King’s College London, Guy’s Campus, London SE1 1UL, UK; b Department of Rheumatology, King’s College London, London, UK; c Academic Department of Surgery, King’s College London, London, UK

**Keywords:** Atherosclerosis, Chemokine, Monocyte, Leukocyte–endothelial adhesion, Cellular adhesion assay

## Abstract

Monocyte adhesion to the arterial endothelium and subsequent migration into the intima are central events in the pathogenesis of atherosclerosis. Previous experimental models have shown that chemokines can enhance monocyte–endothelial adhesion by activating monocyte integrins. Our study assesses the role of chemokines IL-8, MCP-1 and GRO-α, together with their monocyte receptors CCR2 and CXCR2 in monocyte adhesion to human atherosclerotic plaques. In an adhesion assay, a suspension of monocytic U937 cells was incubated with human atherosclerotic artery sections and the levels of endothelial adhesion were quantified. Adhesion performed in the presence of a monoclonal antibody to a chemokine, chemokine receptor or of an isotype matched control immunoglobulin, shows that antibodies to all chemokines tested, as well as their receptors, inhibit adhesion compared to the control immunoglobulins. Immunohistochemistry demonstrated the expression of MCP-1, GRO-α and their receptors in the endothelial cells and intima of all atherosclerotic lesions. These results suggest that all these chemokines and their receptors can play a role in the adhesion of monocytes to human atherosclerotic plaques. Furthermore, they suggest that these chemokine interactions provide potential targets for the therapy of atherosclerosis.

## 1 Introduction

The migration of monocytes into the intima gives rise to a macrophage population that is central to atherosclerosis. It is clearly important to identify accurately in the human disease the adhesion mechanisms that allow monocyte traffic into the arterial wall. There is a possibility that species differences may exist, for example our previous functional study on human tissues [1] failed to confirm a role for VCAM-1, an adhesion molecule implicated in animal models. In this study on human atherosclerosis we investigate the roles of three chemokines and their receptors in inducing monocyte adhesion.

Monocyte–endothelial contact through adhesion molecules can be enhanced by chemokines, which can induce arrest of cells from flow, in addition to their chemotactic role [2,3]. Consequent activation of integrins can mediate tight static adhesion [4]. Adhesion requires higher levels of receptor stimulation than chemotaxis [5].

MCP-1 (Monocyte chemoattractant protein-1, CCL2) acts via its receptor CCR2. GRO-α (Growth related oncogene-α, CXCL1) and IL-8 (Interleukin 8, CXCL8) share a common receptor CXCR2. MCP-1 is a major monocyte chemotactic factor that is synthesized in many cell types. It is induced by modified-LDL in endothelial cells [6], and may trigger firm adhesion of monocytes to vascular endothelium under flow [7], but not in all studies [8,9] It can stimulate macrophage infiltration into the arterial wall [10], but there is little information on its level in the endothelium. Reduction of lesion size in MCP-1^−/−^ apoE^−/−^ mice has implicated it in the apoE gene deleted mouse atherosclerosis model [11]. CCR2 is a G-coupled receptor, through which MCP-1 induces monocyte adhesion and chemotaxis. Sustained adhesion to endothelium may result from a prolonged activation of Mac-1 integrin and binding to ICAM-1 [12]. Gene disruption experiments have implicated it in murine atherosclerosis [13].

GRO-α is induced by oxidised LDL [14] and laminar shear stress [15] in endothelial cells. In mouse atherosclerosis it has a major role in monocyte adhesion [9], but its involvement in the human disease has not been reported. It induces monocyte adhesion to modified-LDL stimulated endothelium [14]. In human umbilical vein endothelial cells (HUVEC) it is induced by TNFα and binds to surface heparan sulphate proteoglycans. This resulted in the firm adhesion of monocytes under flow conditions [8].

IL-8 is associated with acute inflammatory states through its potent neutrophil chemotactic effects. It is induced by oxidised LDL and low shear stress in endothelial cells [16], and has been detected in the endothelium of human atherosclerotic plaques [17]. Like MCP-1 it has been implicated in firm adhesion of monocytes to E-selectin expressing monolayers of vascular endothelium [18].

CXCR2 is the G-coupled receptor of both GRO-α and IL-8. Its expression is proatherogenic as CXCR2 deficiency reduces the progression of advanced atherosclerosis in mice, and it may have a role in retaining macrophages in the lesions [19]. Oxidised LDL upregulates the expression of CXCR2 on the surface of human monocytes, and of its mRNA [20]. Recently, the cytokine MIF (macrophage migration inhibition factor) has been found to have a role in leukocyte recruitment in atherosclerosis by signalling through CXCR2 and CXCR4 [21]. CXCR1 also serves as a receptor for IL-8 and GRO-α on neutrophils, but the levels on human monocytes and macrophages are low [22] and no functional information is available on its role in these cells. It has not been investigated in this study.

Previous investigation of the role of chemokines in a mouse atherosclerosis model showed that KC, the mouse CXCL1, induced the arrest of monocytes on atherosclerotic endothelium in vivo [9]. However, JE, the mouse CCL2, was ineffective. KC was operative through activation of α_4_β_1_ integrin and its binding to endothelial VCAM-1, but such a VCAM-1 dependent mechanism may not apply in man, as mentioned above [1].

The human disease can be investigated by the previously devised ex vivo technique in which sections of human atherosclerotic arteries are tested for adhesion with the U937 human monocytic cell line [1]. We used this assay to investigate the effect of blocking monocyte–endothelial adhesion by antibodies to chemokines and their receptors. We have also confirmed by immunohistochemistry the expression of these molecules in the endothelium of atherosclerotic human carotid arteries.

## 2 Materials and methods

### 2.1 Human carotid endarterectomy specimens

Human carotid endarterectomy specimens were obtained with ethical permission from male or female patients aged between 65 and 80 with a carotid stenosis of greater than 70%, as demonstrated by digital subtraction angiography and duplex ultrasonography. The investigation conformed with the principles outlined in the Declaration of Helsinki [23]. The arteries used had advanced atherosclerotic lesions of grade III–V in Stary classification. The arteries were snap frozen and stored at −80 °C. Cryostat sections from the stored specimens were cut at 8 μm and placed on APES coated slides and dried for 2 h. The slides were stored at −80 °C.

### 2.2 Antibodies

Antibodies to MCP-1 (clone hmcp1, IgG_2a_, Serotec, Kidlington, UK), GRO-α (clone 20326.1, IgG_2b_, Abcam, Cambridge, UK), IL-8 (clone 6217, IgG_1_, R&D Systems, Abingdon, UK), CCR2 (clone 48607.121, IgG_2b_, R&D Systems) and CXCR2 (clone 48311, IgG_2a_, R&D Systems) were used for inhibition of cell adhesion. They were compared with their isotype matched control immunoglobulins UPC10 (IgG_2a_), MOPC141 (IgG_2b_) and MOPC21 (IgG_1_) (Sigma–Aldrich, Gillingham, Dorset, UK) as the negative control. UPC10 has irrelevant antibody activity against β2–6 linked fructosan (supplier’s datasheet). Previous work had shown that these control immunoglobulins have little effect on adhesion themselves [1]. EBM11 (IgG_1_, CD68, Dako) was also used as a negative control. All antibodies and immunoglobulins were added to the cell suspension at 20 μg/ml immediately before addition to the tissue sections. This concentration of antibody had been established as optimal from previous titration experiments.

### 2.3 Cell culture and tissue adhesion assay

U937 cells were cultured and the assay performed essentially as previously described [1]. Briefly, the cultured cells first had the medium pH corrected to neutrality, and then were stimulated with 10 ng/ml PMA for 16–24 h. Native U937 cells are at a promonocytic stage of differentiation, and require stimulation with PMA to enable their development to a monocytic phenotype with expression of CD14. These cells then have adhesive properties on arterial tissue sections that are very similar to those of blood monocytes [1]. They were then washed, antibodies were added, and 200 μl of cell suspension was added to the slides, which were placed on a horizontally rotating tray, and rotated at 60 rpm for 40 min at 37 °C. The slides were then placed vertically in a slide staining rack and rapidly dipped six times into a PBS bath to remove non-adherent cells. They were fixed with 4% paraformaldehyde (VWR, Lutterworth, UK) for 20 min. The endothelium was immunostained with peroxidase conjugated rabbit anti-human von Willebrand Factor (vWF) (Dako) 13 μg/ml for 30 min. They were washed twice, and the reaction developed with hydrogen peroxide and diaminobenzidine (DAB) (Sigma) for about 10 min. Slides were washed in water, counterstained in Mayer’s haemalum (VWR) for 30 s, rinsed, and differentiated for 10 s in acid alcohol (0.5% concentrated HCl in 70% ethanol/water). They were then dehydrated in three baths of 96% ethanol and two of xylene, 2 min in each, and mounted in DPX (VWR). For each antibody assessed, three experiments with quadruplicate slides were performed, each experiment with a different artery. Other adhesion experiments on the properties of control and irrelevant antibodies were done as triplicate assays. Previous work [1] had shown that uninhibited adhesion to normal artery was substantially less than to atherosclerotic plaque, so normal tissue was not investigated in this study.

### 2.4 Cell counting

**Fig. 1:**
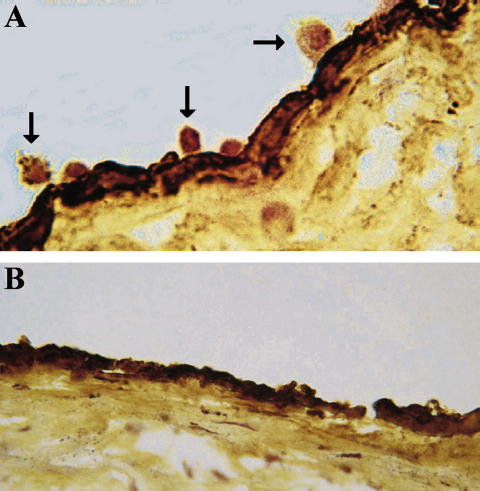
U937 cell–atherosclerotic plaque adhesion assay, performed as described in Section 2. The endothelium is stained with an anti-von Willebrand factor antibody. (A) Uninhibited adhesion with control mouse immunoglobulin 20 μg/ml. U937 cells are adhering to the surface of the endothelium (arrows), and to the cut face of the intima. (B) U937 adhesion is inhibited by the same concentration of mouse anti-GROα antibody. Representative example of 12 experiments, original magnification 630×.

Cells attached to an intact endothelial luminal surface were counted. The counting was performed on an image analyser, and the endothelial segment lengths were measured. Usually 12 segments of endothelium of mean length 170 μm were assessed per slide. Adhesion was expressed as cell numbers/mm of endothelium. Analysis was performed blind to slide conditions. Cells also adhered to the cut surface of the intima (Fig. 1A), as previously reported [1] but these were not quantitated.

### 2.5 Statistical analysis

Results are given as percentage of control adhesion ± standard error. They were analysed by two-way analysis of variance (ANOVA, Prism, Graph Pad Prism Software, San Diego, CA). A *p*-value of <0.05 was considered significant.

### 2.6 Immunohistochemistry

Immunohistochemistry (reagents from Dako) was performed by the avidin–biotin complex (ABC) technique to characterize the arteries and to assess the expression of the chemokines and their receptors. The arteries were first blocked in normal rabbit serum 1/10. Reagents were made in PBS, and stages were separated by two 5 min washes, except after this blocking. Primary antibodies were EBM11 (4.50 μg/ml) for CD68/ macrophages and HHF35 (1 μg/ml) for smooth muscle cell actin, in 90 min incubations. For chemokine/receptor staining (6 arteries), antibodies were used at 20 μg/ml. The secondary antibody was biotinylated F(ab′)_2_ rabbit anti-mouse immunoglobulin (2.1 μg/ml), and was incubated for 30 min. The avidin–biotin–peroxidase complex was then applied for 30 min. The staining was then developed with DAB as described above.

### 2.7 Flow cytometry

U937 cells were stimulated with PMA as described, washed, and incubated at 2.4 × 10^6^ cells/ml with 20 μg/ml of the CCR2 and CXCR2 antibodies for 60 min, then washed twice and incubated with fluorescein labelled F(ab′)_2_ rabbit anti-mouse immunoglobulin (Dako) 20 μg/ml for 30 min. The negative primary antibody control was isotype matched non-immune mouse immunoglobulin (Sigma). The cells were washed twice and read in an EPICS XL flow cytometer (Beckman-Coulter, High Wycombe, UK).

## 3 Results

### 3.1 Specific inhibition of U937 cell adhesion to atherosclerotic human artery endothelium by antibodies to chemokines

#### 3.1.1 MCP-1

**Fig. 2:**
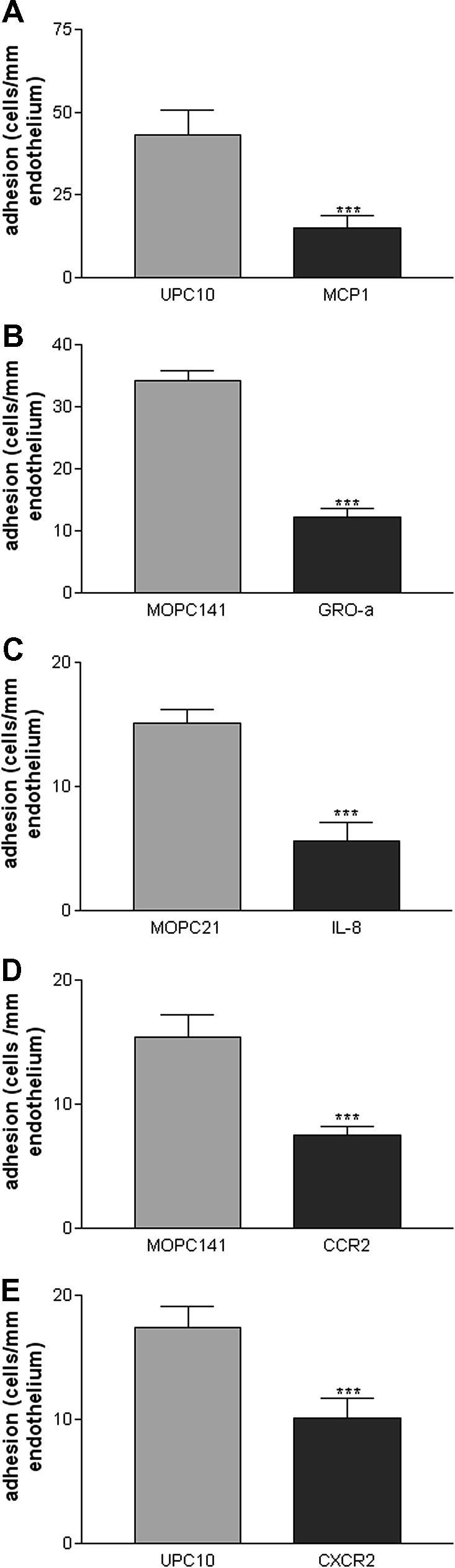
Inhibition of U937 cell–plaque endothelium adhesion by antibodies to chemokines and their receptors. The assays were performed as described in Section 2: each graph shows adhesion in isotype matched control immunoglobulin 20 μg/ml compared to adhesion with antibody 20 μg/ml to the antigen indicated. The mean ± SE of U937 cell adhesion per length of endothelium in three quadruplicate experiments is shown. All antibodies cause highly significant inhibition of adhesion, ^∗∗∗^*p* < 0.001. (A) Anti-MCP-1; (B) anti-GROα; (C) anti-IL-8; (D) anti-CCR-2; (E) anti-CXCR-2.

U937 cells bound to the endothelial cells of the sections of atherosclerotic plaques, as previously described [1] (Fig. 1A), and chemokine antibodies inhibited this adhesion (Fig. 1B). The effect of monoclonal antibody against MCP-1 was assessed by comparison with the control immunoglobulin UPC10. It was shown that MCP-1 blockage in the three experiments performed on three different tissues leads to endothelial adhesion of 34.6 ± 3.5% of the level of the control (*p* < 0.001). The results are represented in Fig. 2A. The control UPC10 immunoglobulin itself gave a mean of 88.0% of control adhesion (not significantly different, NS) compared to tissue culture medium in five experiments. Similarly EBM11, an antibody against the monocyte/macrophage intracellular antigen, CD68, did not inhibit adhesion either to the endothelium (104% of tissue culture control, NS) or the cut surface of the intima (2 experiments).

#### 3.1.2 GRO-α

Three adhesion assays assessing the effect of monoclonal antibody against GRO-α showed an inhibition of monocyte adhesion to 35.6 ± 5.2% of control (*p* < 0.001). Comparison was made with the control immunoglobulin MOPC141. The results are shown in Fig. 2B, and a histological image in Fig. 1B. MOPC141 itself gave 104% (NS) of control adhesion compared to tissue culture medium (triplicate experiment).

#### 3.1.3 IL-8

IL-8 blockage in the three experiments performed on different tissues lead to adhesion at a level of 37.2 ± 9.6% of the control (*p* < 0.001). Comparison was made with the control immunoglobulin MOPC21. The results are represented in Fig. 2C. MOPC21 gave 88.4% of control adhesion (NS) compared to tissue culture medium in eight experiments.

### 3.2 Specific inhibition of U937 cell adhesion to human atherosclerotic plaque endothelium by antibodies to chemokine receptors

#### 3.2.1 CCR2

Three adhesion assays assessing the effect of monoclonal antibody against CCR2 showed an inhibition of monocyte adhesion to 48.6 ± 4.8% of the control with MOPC141 (*p* < 0.001). The results are shown in Fig. 2D.

#### 3.2.2 CXCR2

Similarly three assays with an antibody against CXCR2 showed an inhibition of monocyte adhesion to 58.4 ± 8.7% of the control with UPC10 (*p* < 0.001). The results are shown in Fig. 2E.

### 3.3 Expression of chemokines and their receptors in atherosclerotic human arteries

#### 3.3.1 MCP-1 and CCR2

**Fig. 3:**
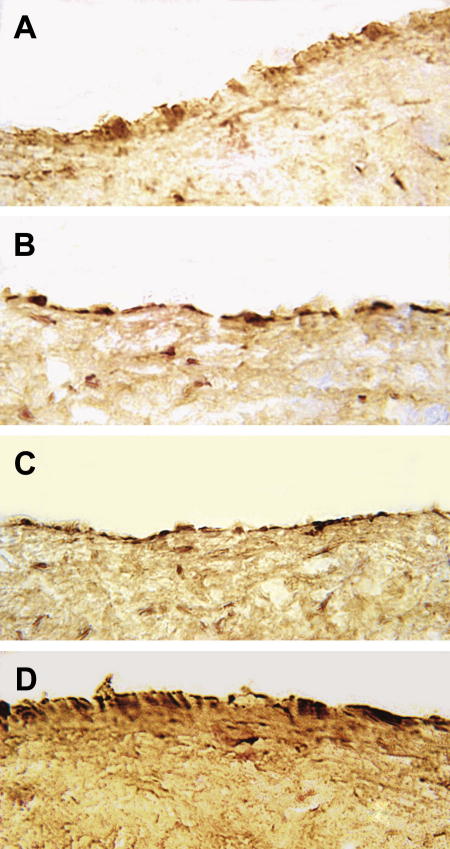
Immunohistochemistry of chemokines and their receptors in atherosclerotic plaque intima. The avidin–biotin complex method was used as described in Section 2, with 20 μg/ml of primary antibody. All chemokines and receptors assayed show strong expression in the surface endothelial cells and lesser reactions in scattered cells within the intima. (A) MCP-1; (B) GROα; (C) CCR-2; (D) CXCR2. Representative examples of 6 arteries shown, original magnification 630×.

Immunohistochemistry shows the presence of both MCP-1 and its receptor CCR2 in the endothelial as well as the intimal cells of advanced atherosclerotic lesions in human carotid arteries (Fig. 3A and C). The expression of MCP-1 is greater in the endothelium overlying atheromatous lesions than in the intima beneath, and in parts is maximal on the apical surface of the endothelium. These findings were consistent in all the atherosclerotic carotid arteries examined.

#### 3.3.2 GRO-α and CXCR2

GRO-α and its receptor CXCR2 are also expressed in both endothelial and intimal cells of advanced human atherosclerotic lesions (Fig. 3B and D). As with MCP-1 and its receptor, the staining is consistently stronger in the endothelium overlying atherosclerotic plaques compared to the connective tissue of their intima. The pattern of CXCR2 expression suggests staining of the apical surface of the endothelium.

### 3.4 Validation of U937 cells as a chemokine receptor expressing monocyte cell model

#### 3.4.1 CCR2 and CXCR2 expression in U937 cells

**Fig. 4:**
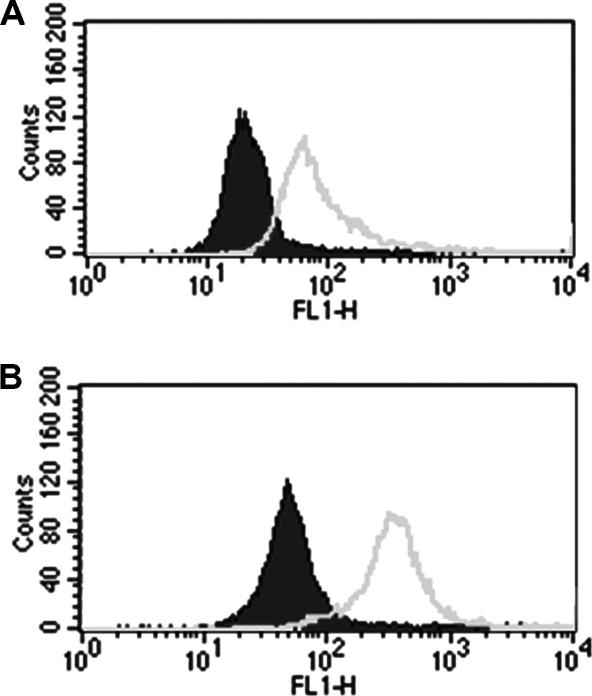
Expression of CXCR-2 in PMA stimulated U937 cells. The cells were stained by a two antibody technique as described in Section 2. Histogram: *X*-axis, fluorescence intensity; *Y*-axis, number of cells. Grey envelope, anti CXCR2; black filled graph, control mouse immunoglobulin UPC10. (A) Surface expression (non-permeabilised); (B) permeabilised cells. CXCR2 is expressed on the U937 cell membrane, and the reaction is enhanced by permeabilisation. One of two similar experiments.

Flow cytometry demonstrated that CXCR2 (Fig. 4A) and CCR2 (not shown) were well expressed on the cell membrane of the PMA stimulated U937 cells used in this study (2 similar experiments). Furthermore, permeabilisation of the cells considerably increased antibody reactivity (Fig. 4B), but with some elevation in background signal.

## 4 Discussion

This study has focused on the ability of chemokines to enhance monocyte–endothelial adhesion in human atherosclerosis. This mechanism is likely to be complementary to their better known chemotactic activity in promoting cell migration into the arterial wall. Our results, of effective blockage of monocyte adhesion to atherosclerotic endothelium by both antibodies to chemokines and to their receptors, provide the first functional evidence for a role of chemokines in inducing monocyte adhesion in human atherosclerosis. These findings are similar to previous studies in animal models. Strong inhibition of adhesion with antibodies to MCP-1, GROα and IL-8 in this assay suggests that all these chemokines have a role in the adhesion of monocytes to the endothelium of human atherosclerotic plaques. The result with CCR2 extends the substantial evidence for its involvement in mouse atherosclerosis [11], but with a possible greater role in monocyte arrest. The findings with GRO-α support and extend the previous evidence for the importance of this cell–surface bound chemokine in inducing monocyte adhesion to mouse atherosclerotic endothelium and to stimulated HUVEC [8,9]. Our finding of a role for IL-8 in the monocyte–endothelial adhesion of atherosclerosis is novel, but is consistent with related findings [18,24]. With the receptors, the results in man with CCR2 parallel its involvement in the mouse [13], as do those for CXCR2 [25].

It is likely that signalling from the chemokines and receptors studied is operating via the activation of β_2_ integrins. Previous work with this assay showed a major role for these integrins in permitting monocyte–endothelial adhesion in human atherosclerosis [1], but equivocal evidence for β_1_involvement (unpublished data).

The chemokine receptors CCR2 and CXCR2 were expressed in the arterial endothelial cells of human atherosclerosis, in addition to their expected expression in the U937 cells, which we have confirmed. These receptors have been noted previously on human endothelial cells [26], and may mediate chemotactic responses involved in the repair of a damaged endothelium. As the U937 cells used in the assay are stimulated with PMA, they may express chemokines, and if so, it is possible that they might interact with the receptors on the atherosclerotic endothelium. Whether such an interaction could contribute to adhesion is not clear. However, such considerations emphasise that PMA stimulated U937 cells model accurately the interactions of activated monocytes in vivo.

This form of adhesion assay with tissue sections was first used in seminal studies by Stamper and Woodruff [27] to demonstrate the interaction of circulating lymphocytes with high endothelial venules in lymph nodes. It enables the efficient use ex vivo of scarce human material, and permits controlled replicated adhesion experiments on the human tissues. Here transverse histological sections of human arteries provide the substrate for adhesion, and allow the comparison of near identical samples in replicates with statistical analysis. As a consequence of the use of tissue sections, the endothelial cells are transected. Therefore, the mode of access of the test U937 cells to the artery wall allows contact with the normally unavailable intracellular contents on the cut face. It is possible that exposed molecules might mediate adhesion interactions not present in vivo, but there is no evidence that this is the case. Furthermore, it is encouraging that the antibody EBM11, against the highly expressed CD68 intracellular antigen of plentiful plaque macrophages, was without effect on adhesion. This suggests that antibody binding to a molecule irrelevant to physiological interactions is without effect. In addition, in this study, non-specific effects of immunoglobulins have been controlled by making comparison with isotype matched non-immune mouse immunoglobulins in all experiments. These immunoglobulins themselves are demonstrated to have no significant effect on adhesion.

From these observations alone it is difficult to determine whether the adhesion ligands operative in the assay are on the apical endothelial surface. However, it is established that MCP-1 [28], GROα [14] and IL-8 are synthesized by endothelial cells, that IL-8 can be transcytosed from the abluminal to the luminal surface [29], and that all chemokines bind to the glycosoaminoglycans of the glycocalyx on the apical surface [30], although MCP-1 may bind less than GRO-α [8]. Interestingly, the MCP-1 immunohistochemistry gives some evidence of endothelial luminal surface staining (Fig. 3A). Therefore, it is very likely that the chemokines studied are presented at the endothelial surface and are capable of enhancing adhesion in vivo.

In the studies on monocyte adhesion done in this laboratory by this technique, antibodies to several monocyte receptors, (e.g. previously β_2_ integrins and CD14; in this report CCR2 and CXCR2), have strongly inhibited the monocyte–endothelial interaction [1]. Antibodies to endothelial adhesion molecules and chemokines (previously P-selectin, ICAM-1; here MCP-1, GROα and IL-8) have acted similarly. This inhibition is not due merely to the presence of antibody on the surface of the cells, as it was shown previously that an antibody to MHC-II, which is expressed strongly on U937 cells, was without inhibitory effect [1].

It is interesting that blocking of so many of the molecules involved in ligand–receptor adhesion interactions can inhibit cellular adhesion. As many molecules are involved, it might be expected that blocking of any one pair would have little effect. The interaction between the various pairs must therefore be complex: sequential operation of ligands and signalling interactions are likely to be involved. Further, as the adhesion mechanism involves multimolecular complexes at focal adhesion contacts, and possibly membrane microdomains such as lipid rafts [31], it is likely that highly organised macromolecular structures are involved, as has been found in the T lymphocyte-antigen presenting cell interaction [32]. These complexes may include chemokine receptors [33]. It seems possible that the molecules are cooperating to produce adhesion in a highly integrated manner, and it may be that ligation or blocking of one can affect the operation of the adhesion mechanism as a whole. Ultrastructural studies could be of interest.

The critical dependence of atherogenesis on monocyte–endothelial adhesion encourages further research on the role of chemokines in its mechanism. The possibility of the use of specific chemokine inhibitors as therapeutic agents in man is supported by this study. Anti-chemokine therapy is effective in animal models [34], and a wide range of established anti-atherosclerotic agents, including aspirin and statins, are already known to inhibit the expression of MCP-1 [35] or its receptor CCR-2 [36].
